# Monoaminergic control of spinal locomotor networks in SOD1^G93A^ newborn mice

**DOI:** 10.3389/fncir.2014.00077

**Published:** 2014-07-04

**Authors:** Léa Milan, Grégory Barrière, Philippe De Deurwaerdère, Jean-René Cazalets, Sandrine S. Bertrand

**Affiliations:** ^1^CNRS, Institut de Neurosciences Cognitives et Intégratives d’Aquitaine, UMR5287, Université de BordeauxBordeaux, France; ^2^Institut des Maladies Neurodégénératives, Université de BordeauxBordeaux, France

**Keywords:** serotonin, dopamine, noradrenaline, spinal cord, ALS, fictive locomotion, HPLC, extracellular recordings

## Abstract

Mutations in the gene that encodes Cu/Zn-superoxide dismutase (SOD1) are the cause of approximately 20% of familial forms of amyotrophic lateral sclerosis (ALS), a fatal neurodegenerative disease characterized by the progressive loss of motor neurons. While ALS symptoms appear in adulthood, spinal motoneurons exhibit functional alterations as early as the embryonic and postnatal stages in the murine model of ALS, the SOD1 mice. Monoaminergic – i.e., dopaminergic (DA), serotoninergic (5-HT), and noradrenergic (NA) – pathways powerfully control spinal networks and contribute significantly to their embryonic and postnatal maturation. Alterations in monoaminergic neuromodulation during development could therefore lead to impairments in the motoneuronal physiology. In this study, we sought to determine whether the monoaminergic spinal systems are modified in the early stages of development in SOD1 mice. Using a post-mortem analysis by high performance liquid chromatography (HPLC), monoaminergic neuromodulators and their metabolites were quantified in the lumbar spinal cord of SOD1 and wild-type (WT) mice aged one postnatal day (P1) and P10. This analysis underscores an increased content of DA in the SOD1 lumbar spinal cord compared to that of WT mice but failed to reveal any modification of the other monoaminergic contents. In a next step, we compared the efficiency of the monoaminergic compounds in triggering and modulating fictive locomotion in WT and SOD1 mice. This study was performed in P1–P3 SOD1 mice and age-matched control littermates using extracellular recordings from the lumbar ventral roots in the *in vitro* isolated spinal cord preparation. This analysis revealed that the spinal networks of SOD1^G93A^ mice could generate normal locomotor activity in the presence of NMA-5-HT. Interestingly, we also observed that SOD1 spinal networks have an increased sensitivity to NA compared to WT spinal circuits but exhibited similar DA responses.

## INTRODUCTION

Amyotrophic lateral sclerosis (ALS) is a fatal neurodegenerative disease characterized by the progressive degeneration of M1 neurons in the cerebral cortex and motor neurons in the brainstem and spinal cord. Approximately 20% of familial forms of ALS cases are associated with inherited dominant mutations in the gene that encodes Cu/Zn-superoxide dismutase (SOD1). While ALS syndrome occurs during adulthood in both humans and ALS animal models, a growing body of evidence shows that spinal locomotor networks exhibit functional alterations as early as the embryonic and postnatal stages in mice expressing the human mutated SOD1 protein, the SOD1 mouse model. Behavioral tests performed during the first postnatal week revealed a delay in the maturation processes of sensorimotor modalities such as righting and hind-paw grasping responses in mutant SOD1^G85R^ mice (Amendola et al., 2004). At the cellular level, developing SOD1^G85R^ and SOD1^G93A^ motoneurons have been shown to exhibit dendritic abnormalities and alterations in both excitability and synaptic inputs compared to the motoneurons of age-matched wild-type (WT) mice (Bories et al., 2007; Pambo-Pambo et al., 2009; Chang and Martin, 2011; Filipchuk and Durand, 2012; Martin et al., 2013). Differences in neurochemical sensitivity have also been reported between newborn WT and SOD1 mice. Indeed, the classical rhythmogenic drug cocktail (a mixture of glutamate agonist plus serotonin) used to activate the locomotor central pattern generators (CPGs) in the isolated spinal cord preparation of newborn rodents has been shown to be inefficient in inducing fictive locomotion in SOD1^G85R^ mice (Amendola et al., 2004). As such early alterations could prime neuronal circuits and make them more permissive to pathological changes later in life, it appears of importance to further decipher the changes undergone by the spinal motor networks in early developmental stages in the SOD1 models of ALS.

Motoneurons are targeted by numerous extra- and intraspinal neuromodulatory systems that control both their intrinsic membrane properties and incoming synaptic inputs (for review see Miles and Sillar, 2011). In addition to their role in excitability control, neuromodulatory influences contribute significantly to the embryonic and postnatal development of the spinal cord motor networks (for review see Vinay et al., 2002; Miles and Sillar, 2011). Amongst neuromodulatory systems, monoaminergic – i.e., dopaminergic (DA), serotoninergic (5-HT), and noradrenergic (NA) – pathways have been shown to initiate and facilitate the expression of spinal motor outputs, to control segmental reflexes and to play a major role in the maturation of spinal locomotor networks (Barbeau and Rossignol, 1991; Crick and Wallis, 1991; Cazalets et al., 1992; Sqalli-Houssaini et al., 1993; Kiehn and Kjaerulff, 1996; Sqalli-Houssaini and Cazalets, 2000; Whelan et al., 2000; Vinay et al., 2002; Barrière et al., 2004, 2008; Han et al., 2007; Han and Whelan, 2009; Tartas et al., 2010; Miles and Sillar, 2011; Humphreys and Whelan, 2012; Pearlstein, 2013). Alterations in monoaminergic controls during development could therefore lead to significant reorganizations of the spinal locomotor circuits and contribute to the developmental impairments described in SOD1 motoneurons. However, to the best of our knowledge, data concerning the possible changes in the spinal monoaminergic inputs in the early developmental stages of SOD1 mice are not currently available. In the present study, we sought to determine whether spinal monoaminergic content and sensitivity is modified in newborn SOD1 mice. Specifically, we performed a high performance liquid chromatography (HPLC) analysis of the spinal monoamine contents and compared the effects of 5-HT, DA, and NA on the locomotor activity recorded extracellularly from isolated spinal cord preparations from newborn SOD1 and age-matched control littermates. This study found that well-organized locomotor-like activity could be generated in the isolated spinal cord preparation from SOD1^G93A^ mice by classical pharmacological activation. We also reported an increased content of spinal DA content in the second postnatal week, as well as an increased sensitivity to NA, in SOD1 spinal networks compared to those of WT mice.

## MATERIALS AND METHODS

### ETHICS STATEMENTS AND ANIMALS

All procedures were conducted in accordance with the local ethics committee of the University of Bordeaux and the European Committee Council Directive. All efforts were made to minimize animal suffering and to reduce the number of animals used in the experiments. Transgenic mice expressing a mutated human SOD-1 gene from the B6SJL-TgN (SOD1^G93A^) 1 Gur/J line (SOD1 mice) were purchased from Jackson Laboratories. These mice were bred in our laboratory and maintained as hemizygotes by mating transgenic males with wild-type B6SJL females. Two hundred thirty-eight newborn mice were used in this study. All of the experiments and analyses presented here have been performed blind to the genotype of the animals. Mice were genotyped from genomic DNA purified from tail biopsies by PCR using the following primers: 5′ CATCAGCCCTAATCCATCTGA 3′ (forward), 5′ CGCGACTAACAATCAAAGTGA 3′ (reverse).

### TISSUE PROCESSING FOR POST-MORTEM ANALYSIS

After decapitation, the lumbar spinal cord of postnatal 1 (P1) or P10 SOD1 and age-matched control littermate male mice was quickly removed by a laminectomy, placed in dry ice and stored at -80^∘^C until experiment processing. When needed, the lumbar spinal cord was placed on the side and cut in the middle of the dorso-ventral axis with micro dissecting knives to separate the ventral and dorsal part of the cord.

### POST-MORTEM HIGH-PERFORMANCE LIQUID CHROMATOGRAPHY (HPLC) MEASUREMENTS

On the day of the biochemical analysis, after weighing the samples, tissue was homogenized in 0.1 N perchloric acid (HClO_4_), sonicated and centrifuged at 13,000 rpm for 30 min at 4^∘^C. The tissue contents of monoamines (DA, NA, 5-HT) and their metabolites (5-hydroxyindole-3-acetic: 5-HIAA a 5-HT metabolite and 3,4-dihydroxyphenylacetic acid: DOPAC, a DA metabolite) were measured by a sensitive HPLC-electrochemical detection (ECD) system. Aliquots of the sample supernatant were placed in an automated autosampler (Shimadzu, SIL-20A, Paris, France) at 4^∘^C to be injected into the HPLC column (Hypersyl C18, 150 mm × 4.6 mm, 5 μm; C.I.L.-Cluzeau, Sainte-Foy-La-Grande, France) protected by a Brownlee–Newguard precolumn (RP-8, 15 × 3.2 mm, 7 μm; C.I.L.-Cluzeau). The mobile phase was delivered at a flow rate of 1.3 ml/min using a HPLC pump (LC20-AD, Shimadzu, France) and was composed as follows (in millimoles): 60 NaH_2_PO_4_, 0.1 disodium EDTA and 2 octane sulfonic acid plus 7% methanol, adjusted to a pH of 3.9 with orthophosphoric acid and filtered through a 0.22 mm Millipore filter. Monoamines and their metabolites were detected using a coulometric cell (Analytical cell 5011, Coulochem) coupled to a programmable detector (Coulochem II, ESA, Paris, France). The potential of the electrodes was set at +350 mV for the oxidation and -270 mV for the reduction. Output signals were recorded on a computer (Beckman, system GOLD). Under these conditions, the sensitivity for NA, DA, 5-HT, DOPAC, and 5-HIAA was 3, 1, 8, 6, and 5 pg/10 μl, respectively, with a signal/noise ratio of 3:1. The tissue content of monoamines was expressed in pg/mg of tissue and corresponded to the mean ± SEM values in each group. Differences of the monoamine content between P1/P10 SOD1 mice and control littermates were analyzed using repeated measures two-way analyses of variance (ANOVA) with Sidak’s multiple comparison tests (Graph Pad Prism). Statistical significance was set at *p* < 0.05.

### ISOLATED SPINAL CORD PREPARATION

Newborn SOD1 and WT littermate mice aged P1–P3 were deeply anesthetized with 4% isoflurane, decapitated and eviscerated. A laminectomy was then performed to expose and remove the spinal cord. All dissections and recording procedures were performed under continuous superperfusion with artificial cerebrospinal fluid (aCSF) containing (in milimoles): NaCl 130, KCl 3, CaCl_2_ 2.5, MgSO_4_ 1.3, NaH_2_PO_4_ 0.58, NaHCO_3_ 25, and glucose 10, with a pH of 7.4 when bubbled with 95% O_2_ + 5% CO_2_ at room temperature (24–26^∘^C).

### PHARMACOLOGY

All drugs (*N*-methyl-D, L-aspartate: NMA; 5-HT, DA, and NA) were obtained from Sigma (St. Louis, MO, USA). Stock solutions of 5-HT and NMA were prepared at 0.1 mM in distilled water and stored at -20^∘^C. Fresh drug solutions of DA and NA were prepared daily and protected from light exposure. Pharmacological compounds were bath-applied using a peristaltic pump (flow rate 7 ml/min).

### EXTRACELLULAR RECORDINGS AND ANALYSIS

Motor outputs were recorded extracellularly from the lumbar ventral roots using glass suction electrodes. In each *in vitro* spinal cord preparation, motor outputs from the right and left lumbar 2 (rL2, lL2, respectively) and one L5 ventral root were simultaneously recorded to investigate both the bilateral segmental alternation and the flexor/extensor activity (Cazalets et al., 1992; Kiehn and Kjaerulff, 1996). The neurograms were amplified (×10000) using high impedance AC amplifiers (200–3000 Hz) built at the laboratory and digitized at 5 kHz (Axograph, Sydney, NSW, Australia) for future analysis. Pharmacologically induced locomotor rhythms were analyzed using non-stationary analysis techniques in a Matlab-based software developed at the laboratory. This custom-made software is similar to SpinalCore, a software developed by the Lev-Tov group (Mor and Lev-Tov, 2007), and is based on the MatLab wavelet coherence package provided by Aslak Grinsted (; Torrence and Compo, 1998; Grinsted et al., 2004). Two-minute sections of pairs of neurograms were analyzed using cross wavelet transform and wavelet coherence. These methods were applied to high-pass (50 Hz), rectified and low-pass filtered (5–10 Hz) signals. For convenience, cross wavelet spectrum and wavelet coherence maps were combined into a mixed cross/coherence map (**Figure 2B1**) highlighting coherent, common high power frequency regions. In these maps, the evolution of the frequency components of the extracellular signals (*y*-axis, logarithmic scale) is represented as a function of time (*x*-axis), and the power of each frequency is color-coded with warm colors assigned to high power regions and cool colors to low power regions. The asymptotic lines in the mixed cross/coherence maps indicate the cone of influence. This cone delimits the region where edge effect becomes too important. The values outside of this cone were thus excluded from the statistical analysis (Torrence and Compo, 1998; Mor and Lev-Tov, 2007). The high power band of the generated time/frequency map was selected as a region of interest (ROI) and arbitrarily segmented into 1 s bins to compute the mean frequency, coherence, and phase relationship between pairs of neurograms. The critical level of statistical significance of the wavelet coherence was calculated using Monte-Carlo simulations (Mor and Lev-Tov, 2007). The power and phase of the mean vector of pairs of ventral root recordings were extracted for each experiment with this procedure. Using the Igor Pro software (Wavemetrics), angular distribution tests and Watson nonparametric circular two sample U2 tests were performed to compare circular data between WT and SOD1 mice in the different pharmacological conditions tested.

Motor burst amplitudes were computed in a custom-made Matlab-based software. For each preparation, the burst amplitude values were normalized to the amplitude measured in the presence of 10 μM 5-HT for experiments with increasing doses of 5-HT or to the amplitude observed during bath-applications of NMA-5-HT (16 μM each; Sqalli-Houssaini et al., 1993) alone prior to the addition of NA or DA to the bath. Repeated measures two-way analyses of variance (ANOVA) with Sidak’s multiple comparison tests were performed to evaluate monoamines and mouse genotype effects (Graph Pad Prism). All data are expressed as means ± SEM. Asterisks in the Figures and Tables indicate positive significance levels of *post hoc* analysis (*p* < 0.05).

## RESULTS

### SPINAL MONOAMINERGIC CONTENTS IN NEWBORN WILD-TYPE AND SOD1 MICE

**FIGURE 1:**
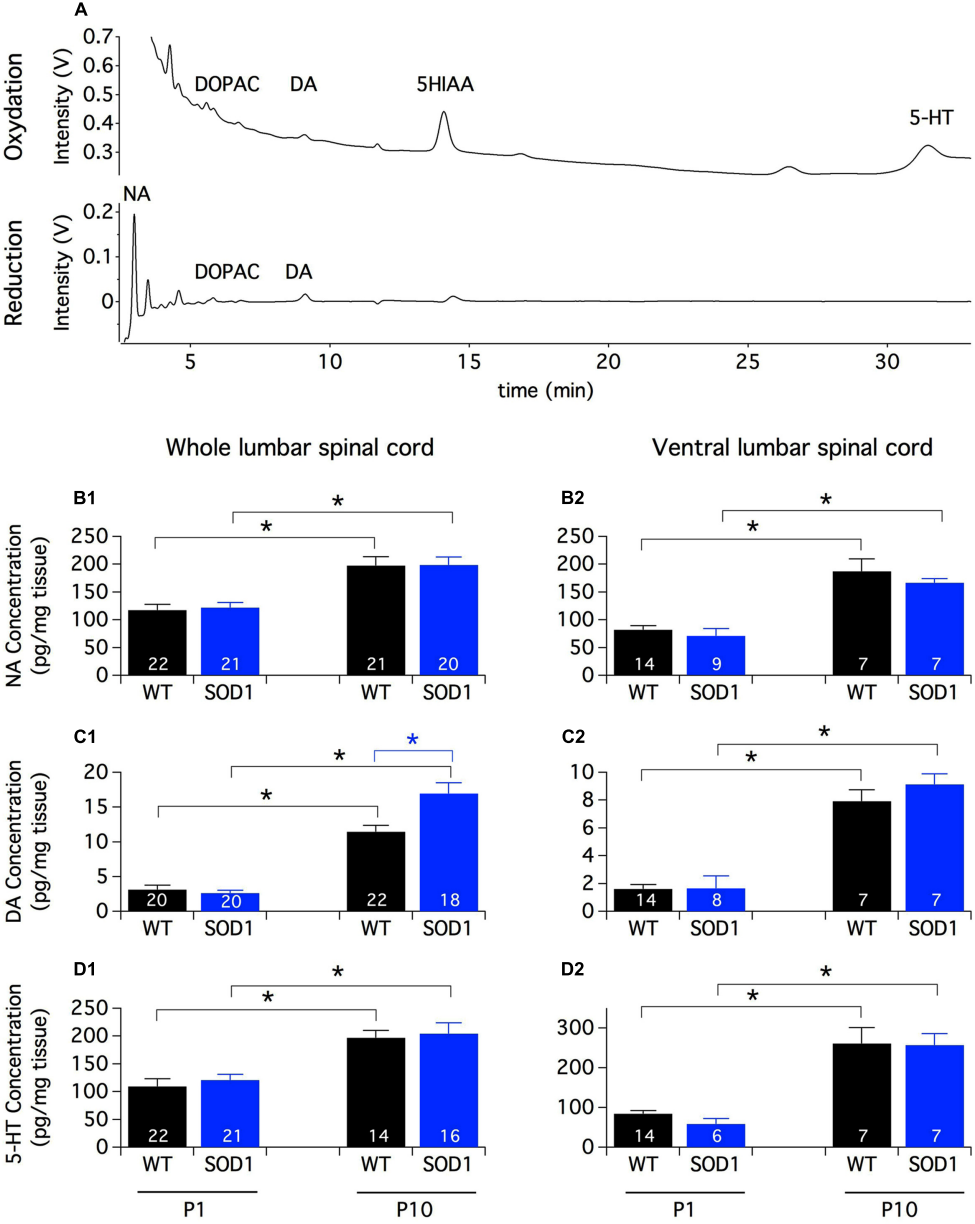
**Monoamine contents in the lumbar spinal cord of newborn mice. (A)** Example of HPLC chromatograms of a whole spinal cord sample from a P10 SOD1 mouse. The chromatograms represent the output Coulometric signals (nanoampere converted in ±1 V output by the recorder) produced after their separation by the compounds at the level of the electrode of oxidation (upper trace; +350 mV) and/or the electrode of reduction (lower trace; -270 mV) of the Coulometric cell. Most of the quantitative analyses are performed using the oxidation channel (all compounds of interest except NA which is often confounded in the solvent front) and NA, DOPAC and DA quantities can be also analyzed using the reduction channel. **(B–D)** Contents of NA **(B1,B2)**, DA **(C1,C2)**, and 5-HT **(D1,D2)** measured by HPLC assays in the whole lumbar spinal cord **(B1–D1)** or in its ventral half **(B2–D2)** from P1 and P10 WT (black bars) and SOD1 (blue bars) mice. Note the increased concentration of monoamines with age and the significant difference in DA content between WT and SOD1 P10 mice. Asterisks indicate positive significance levels and the numbers in histogram bars refer to the number of samples tested. NA: noradrenaline, DA: dopamine, 5-HT: serotonin, DOPAC: 3,4-dihydroxyphenylacetic acid, 5-HIAA: 5-hydroxyindole-3-acetic.

In rats, the first connections between brainstem monoaminergic cells and spinal neurons are established during the last week of gestation and mature sequentially along the rostrocaudal and ventro-dorsal axes until the second postnatal week (Commissiong, 1983; Bregman, 1987; Rajaofetra et al., 1989, 1992; Giménez y Ribotta et al., 1998; Clarac et al., 2004). To get an overview of the spinal monoaminergic innervation, we first conducted an HPLC analysis of the endogenous spinal content of biogenic amines and their metabolites in SOD1 mice and WT littermates (**Figure 1A**). To assess the impact of the descending pathway maturation on monoamine contents, we performed these procedures on the lumbar spinal cord samples from both P1 and P10 animals. This HPLC analysis revealed that the NA and 5-HT contents were about 20 times greater than the DA content in the lumbar spinal cord of both P1 and P10 mice. We also observed that regardless of the mouse genotype, the contents of NA (**Figure 1B1**), DA (**Figure 1C1**) and 5-HT (**Figure 1D1**) were significantly higher in P10 animals compared to P1 mice. In contrast, DOPAC and 5-HIAA were not significantly different between the two developmental stages tested (**Table 1**). *Post hoc* pairwise comparisons conducted between WT and SOD1 mice revealed no significant change in NA, 5-HT, DOPAC, or 5-HIAA. However, we observed that the DA content was significantly enhanced in the whole lumbar spinal cord of SOD1 P10 mice compared to age-matched WT animals.

**Table 1:** HPLC measurements of 3,4-dihydroxyphenylacetic acid (DOPAC) and 5-hydroxyindole-3-acetic (5-HIAA) contents in the whole or ventral part of the lumbar spinal cord from P1 and P10 WT and SOD1 mice.

		Wild-type		SOD1	
		P1	P10	P1	P10
DOPAC	Whole lumbar	14.3 ± 4 (14)	27.8 ± 3 (18)	33.4 ± 10 (17)	26.9 ± 4 (17)
	Ventral lumbar	8.8 ± 1 (13)	28.2 ± 8 (7)*	11.5 ± 3 (6)	34.8 ± 9 (7)*
5-HIAA	Whole lumbar	148.9 ± 14	174.4 ± 18	154.7 ± 11	176.1 ± 17
	Ventral lumbar	99.4 ± 11	122.8 ± 15	100.9 ± 22	128.7 ± 19

*The numbers in brackets correspond to the number of samples tested. Asterisks indicate significant differences between the P1 and P10 developmental stages.*

Monoaminergic pathways densely innervate both dorsal and ventral spinal circuits. To specifically look for changes in the monoaminergic contents in spinal motor networks, the same HPLC analysis was repeated on the ventral half of the lumbar spinal cord (**Figures 1B2,C2,D2**). In these experimental conditions, we observed that the contents of all of the monoaminergic compounds tested (**Figures 1B2,C2,D2**) except 5-HIAA (**Table 1**) significantly increased during development. In contrast to the aforementioned results obtained using the whole spinal cord, the mouse genotype had no detectable influence on the monoaminergic contents measured in the ventral part of the cord.

### RHYTHMOGENIC CAPABILITIES OF SOD1^G93A^ SPINAL NETWORKS AND SEROTONINERGIC MODULATION

**FIGURE 2:**
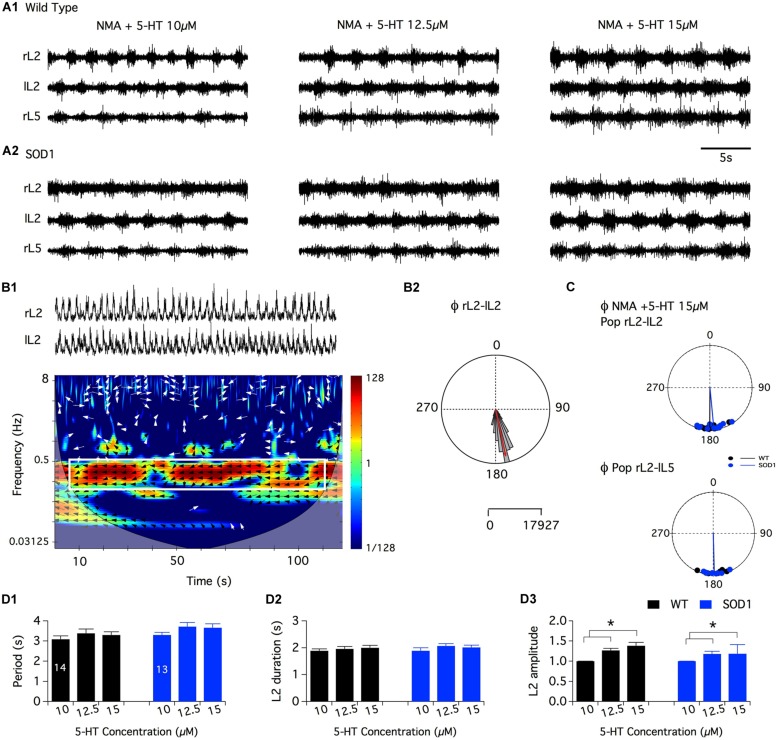
**Serotoninergic modulation of SOD1^**G93A**^ spinal locomotor networks. (A)** Representative traces of the fictive locomotion recorded from the right and left lumbar 2 (rL2 and lL2) and right lumbar 5 (rL5) ventral roots in the presence of 16 μm NMA and increasing concentrations of serotonin in WT **(A1)** and SOD1 **(A2)** newborn mice. **(B)** Mixed cross-coherence time frequency map **(B1)** computed from the high pass, rectified and low pass filtered traces presented in the upper panel recorded in the presence of 16 μm NMA+ 15 μm 5-HT. The white box on the map represents the region of interest selected for further analysis. Black and white arrows indicate phase relationships with a left direction indicating an out of phase relationship. Polar graph **(B2)** of the phase (Φ) relationships between rL2 and lL2 extracted from the graph in **B1**. Calibration bars indicate the number of values in the largest histogram of repartition (5^∘^bins). The evolution of the frequency components of the extracellular signals (*y*-axis, logarithmic scale) is represented as a function of time (*x*-axis). The power of the frequency is color-coded with warm colors assigned to high power regions and cool colors to low power regions. **(C)** Polar graphs of the mean phase relationships between rL2 and lL2 (upper panel) and rL2-lL5 (lower panel) for all the WT (black dots and line, n = 14) and SOD1 (blue dots and line, n = 13) spinal cord preparations tested in the presence of NMA+5-HT 15 μm. Pop: population. **(D)** Plots of the period **(D1)**, L2 burst duration **(D2)** and amplitude **(D3)** in the presence of increasing concentrations of 5-HT in WT (black bars) and SOD1 (blue bars) mice. This analysis reveals that SOD1 spinal cords exhibit a WT-like serotoninergic sensitivity. Asterisks indicate positive significance levels and the numbers in histogram bars refer to the number of spinal cord preparations tested.

As previously mentioned, Amendola et al. (2004) reported that NMA (10–20 μM) plus 5-HT (5–20 μM), a mixture known to generate locomotor-like activity in the *in vitro* spinal cord preparation of newborn rodents, induces only tonic activity when bath-applied to SOD1^G85R^ mouse spinal networks (Amendola et al., 2004). Dramatically different alterations in the motoneuronal physiology have been reported depending on the SOD1 mouse model used (see for example Meehan et al., 2010; Delestrée et al., 2014). The question then arises as to whether NMA-5-HT effects are similar in the SOD1^G85R^ and SOD1^G93A^ high expressor line mouse strains. To address this question, we compared the efficiency of NMA (16 μm) in the presence of increasing concentrations of 5-HT in activating the spinal locomotor CPGs in SOD1^G93A^ and age-matched littermate controls. **Figure 2A** shows that, regardless of the 5-HT concentration tested, the bath-application of NMA-5-HT triggered an alternating bursting activity of the right and left L2 and homolateral flexor L2 and extensor L5 ventral roots in both WT (**Figure 2A1**) and SOD1 mice (**Figure 2A2**). This motor pattern, with a mean period ranging from 3 to 4 s (**Figure 2D1**), is characteristic of a locomotor-like activity (Cazalets et al., 1992; Kiehn and Kjaerulff, 1996). Regardless of the animal genotype, raising the 5-HT concentration from 10 to 12.5 or 15 μm did not significantly affect the rhythm phase relationships (**Figures 2B,C**; **Table 1**), period (**Figure 2D1**) or motor burst duration (L2: **Figure 2D2**, Data not shown for L5). In contrast, the locomotor burst amplitude was significantly increased in the presence of 12.5 or 15 μm 5-HT compared to the 10 μm 5-HT condition (**Figure 2D3** for L2 bursts, data not shown for L5). This 5-HT-induced amplification was, however, not significantly different between the WT and SOD1 mice.

Overall, these data suggest that the spinal networks of newborn SOD1^G93A^ mice could generate well-organized locomotor patterns under NMA-5-HT chemical stimulation and present a WT-like serotoninergic sensitivity.

### NORADRENERGIC MODULATION

**FIGURE 3:**
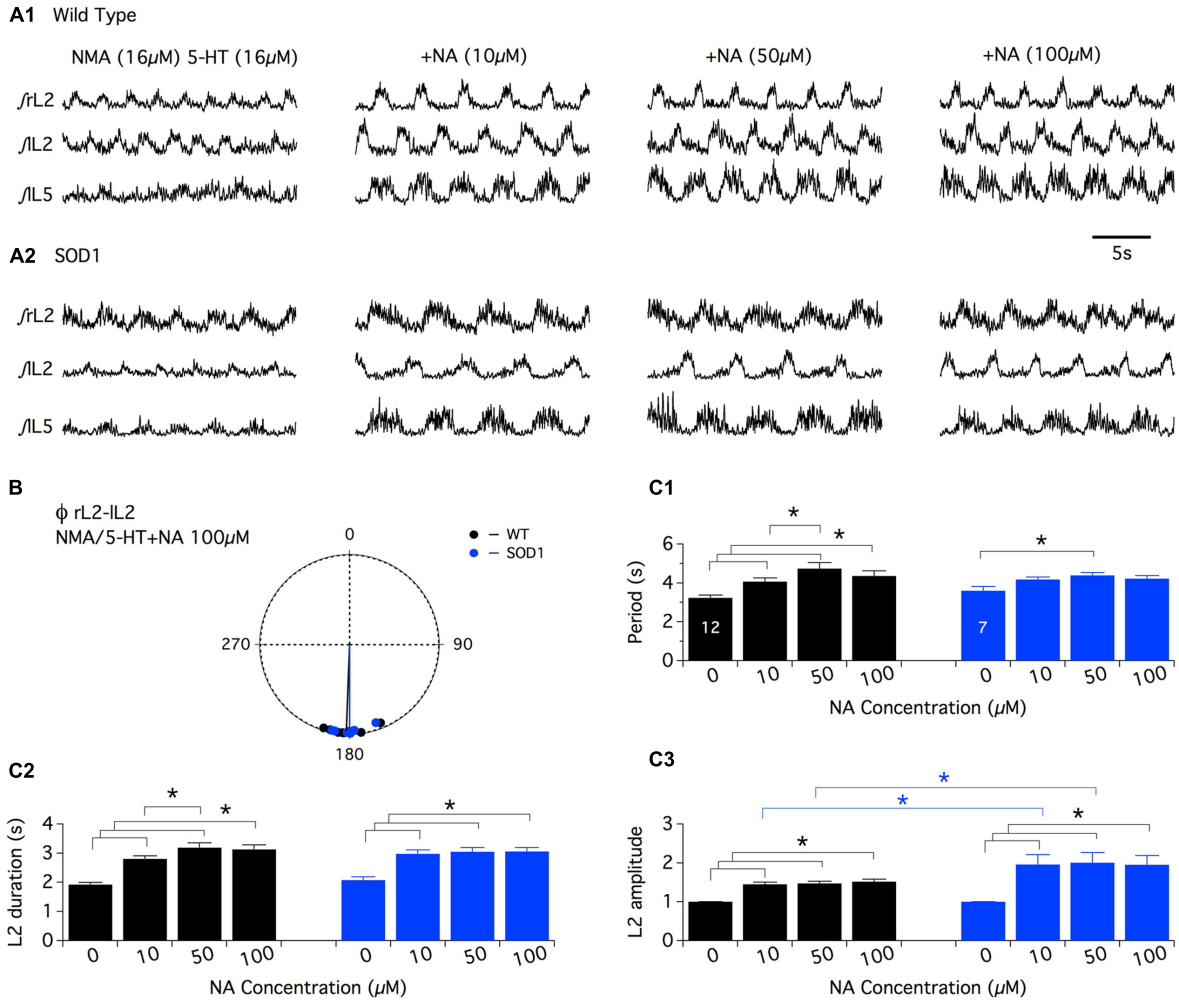
**Neuromodulatory actions of noradrenaline on NMA+5-HT-induced fictive locomotion in SOD1 and age-matched control mice. (A)** Representative integrated (∫ ) extracellular recordings from the right, left L2 and left L5 ventral roots (rL2, lL2, and lL5) in the presence of NMA+5-HT (16 μm each) alone or with noradrenaline (NA; 10, 50, or 100 μm) in WT animals **(A1)** and SOD1 mice **(A2)**. **(B)** Polar graph of the rL2–lL2 phase (Φ) relationships computed in WT (black dots and line, *n* = 12) and SOD1 (blue dots and line, *n* = 7) spinal cords in the presence of 100 μm NA. **(C)** Plots of the period **(C1)**, L2 burst duration **(C2)** and amplitude **(C3)** in the absence or presence of NA bath-applied at increasing concentrations on WT (black bars) and SOD1 (blue bars) spinal cord preparations. Note the significant difference in L2 motor burst modulation between WT and SOD1 mice. Asterisks indicate positive significance levels and the numbers in histogram bars refer to the number of spinal cord preparations tested.

In a first series of experiments, the effects of the bath-application of NA alone (10^-5^, 5 × 10^-^5, and 10^-4^ M (Sqalli-Houssaini and Cazalets, 2000) or in combination with NMA were tested. Under these conditions, tonic or very irregular motor activities were generated in both WT and SOD1 preparations, preventing accurate measurements from being performed (data not shown). In this context, we decided to restrict the analysis of the noradrenergic effects to the modulation of the NMA-5-HT-induced rhythm. After inducing control fictive locomotion with NMA-5-HT (16 μm each), WT and SOD1 spinal cords were challenged with increasing NA concentrations in the presence of NMA-5-HT (**Figure 3**). Regardless of the NA concentration and mouse genotype, the phase relationships of the NA+NMA/5-HT-induced rhythms were similar to those computed in the absence of NA (**Table 2**; **Figure 3B**; Data not shown for the L2/L5 alternation). In contrast, the locomotor rhythm was strongly slowed down in the presence of NA (**Figure 3A**). In WT animals (**Figure 3A1**), all of the NA concentrations tested (10, 50, and 100 μm) induced a significant increase in the locomotor parameters (period, **Figure 3C1**; L2 burst duration, **Figure 3C2**, and amplitude, L2: **Figure 3C3**, black bars, L5: data not shown) compared to the NMA-5-HT control conditions. The rhythm period and L2 burst duration values computed in the presence of 50 μm NA were also significantly different from the ones obtained with 10 μm NA in WT animals. In SOD1 mice (**Figure 3A2**), both the L2 burst durations and amplitude values were significantly enhanced compared to the NMA-5-HT condition for all of the NA concentrations tested (**Figures 3C2,C3**, blue bars). In contrast, the locomotor rhythm period was only significantly different from the control condition in the presence of 50 μm NA (**Figure 3C1**).

**Table 2:** Circular statistics of the phase relationships between right and left L2s in the presence of the different monaminergic concentrations.

	NMA 16 μm					
	+ 5-HT 10 μm		+ 5-HT 12.5 μm		+ 5-HT 15 μm	
	WT	SOD1	WT	SOD1	WT	SOD1
Mean vector	0.901	0.875	0.893	0.89	0.942 (14)	0.924 (13)
Mean angle	-3.11	3.11	-3.12	2.99	-3.13	3.04

	**NMA + 5-HT**		+ **DA 50**μ**m**		+ **DA 500 μm**	
	**WT**	**SOD1**	**WT**	**SOD1**	**WT**	**SOD1**
Mean vector	0.893	0.876	0.960	0.954	0.957 (16)	0.910 (11)
Mean angle	-3.09	2.98	3.02	-3.10	3.02	-3.02

	**NMA + 5-HT**		+ **NA 10 μm**		+ **NA 50 μm**		+ **NA 100 μm**	
	**WT**	**SOD1**	**WT**	**SOD1**	**WT**	**SOD1**	**WT**	**SOD1**
Mean vector	0.950	0.931	0.971	0.975	0.968	0.964	0.961 (12)	0.954 (7)
Mean angle	-3.09	3.03	3.13	3.08	3.06	3.08	-3.105	3.139

*16 μm NMA and 16 μm 5-HT were used to induce control locomotor-like activity. The number of preparations tested in each condition is indicated in brackets. Circular two sample test analysis revealed no significant differences between the experimental conditions tested.*

Interestingly, a *post hoc* analysis revealed that the NA-induced amplification of the L2 (**Figure 3C3**) and L5 (data not shown) burst amplitude values observed in the presence of 10 and 50 μm NA was significantly higher in SOD1 mice compared to WT animals. This result underscores the differences in the NA sensitivity between WT and SOD1 spinal locomotor networks.

### DOPAMINERGIC MODULATION

**FIGURE 4:**
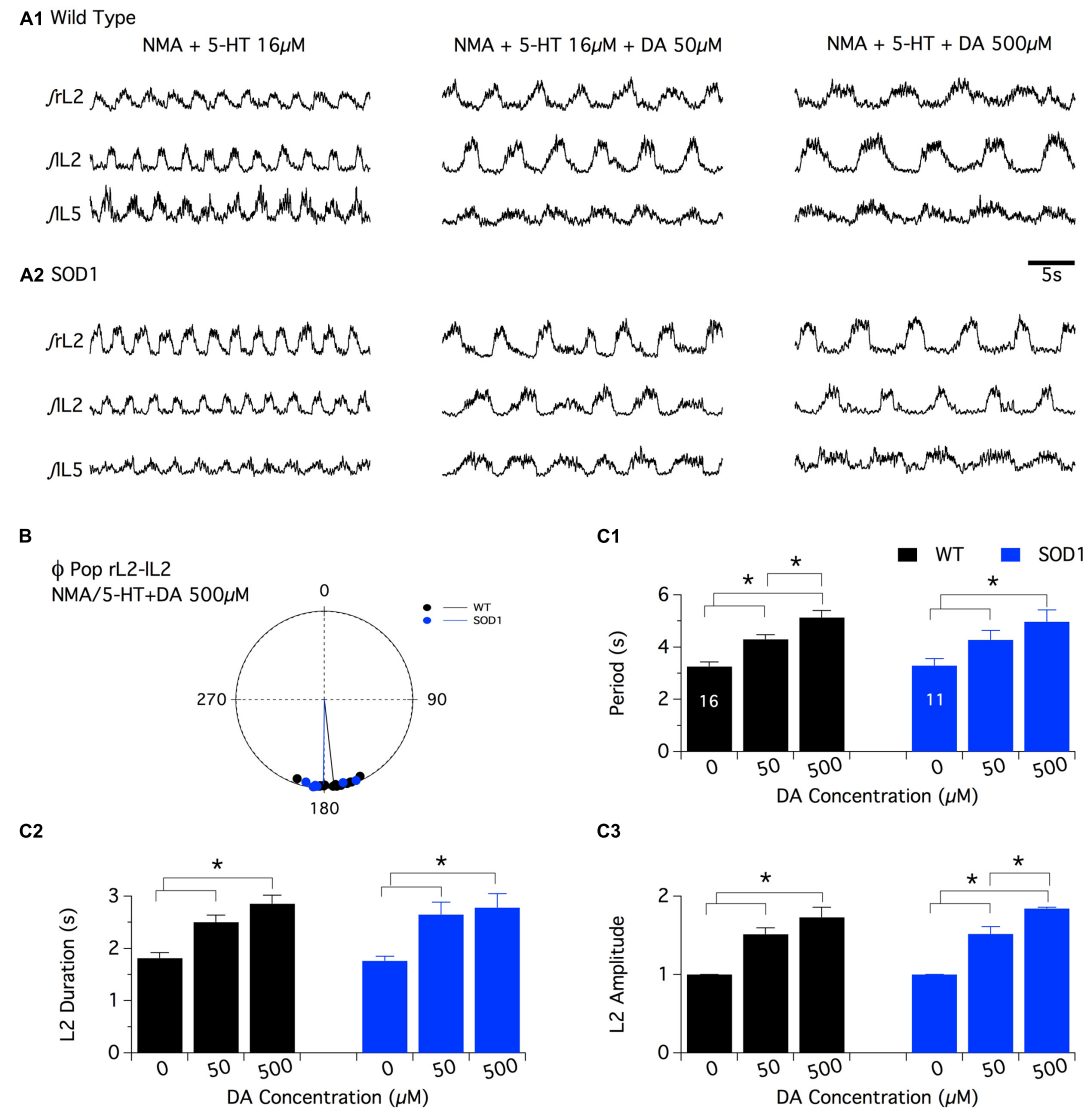
**Effects of dopamine on NMA+5-HT-induced fictive locomotion in SOD1 and age-matched control mice. (A)** Representative integrated (∫ ) traces of NMA + 5-HT (16 μm each)-induced fictive locomotion recorded extracellularly from the right, left L2 and left L5 ventral roots (rL2, lL2, and lL5) in the absence or presence of dopamine (DA, 50 or 500 μm). Upper panels present traces obtained in WT animals **(A1)** and lower panels in SOD1 mice **(A2)**. **(B)** Polar graph of the rL2-lL2 phase (Φ) relationships computed in WT (black dots and line, *n* = 16) and SOD1 (blue dots and line, *n* = 11) spinal cords in the presence of 500 μm DA. **(C)** Plots of the period **(C1)**, L2 burst duration **(C2)** and amplitude **(C3)** in the absence (NMA + 5-HT alone) or presence of increasing concentrations of DA in WT (black bars) and SOD1 (blue bars) mice. These data show that the SOD1 genotype has no effect on the DA neuromodulation when investigated at the extracellular level. Asterisks indicate positive significance levels and the numbers in histogram bars refer to the number of spinal cord preparations tested.

As previously described for NA, the superfusion of DA by itself (10^-4^ and 5 × 10^-4^ M) or in combination with NMA (DA: 10^-5^ M, 5 × 10^-5^ M and 10^-4^ M (Barrière et al., 2004; Spalloni et al., 2011) on *in vitro* spinal cord preparations failed to generate regular motor activities that could be accurately analyzed in both WT and SOD1 preparations (data not shown; see also (Jiang et al., 1999). The comparison of the dopaminergic modulation between WT and SOD1 spinal cord networks was therefore restricted to the NMA-5-HT-induced locomotor like activity. For this purpose, control fictive locomotion was first acquired in the presence of NMA-5-HT (16 μm each, **Figure 4A**) and subsequent DA bath-applications of increasing concentration (50 and 500 μm) were realized. Similar to the other two amines tested, DA did not affect the phase relationships of the NMA-5-HT-induced locomotor activity (**Table 2** and **Figure 4B**, data not shown for the L2/L5 alternation) in both WT and SOD1 spinal cord preparations. However, irrespective of the mouse genotype, 50 or 500 μm DA significantly increased the values of all locomotor parameters (period, **Figure 4C1**; L2 burst duration, **Figure 4C2** and burst amplitude values, **Figure 4C3**, data not shown for L5) compared to the control NMA-5-HT condition. The period of the locomotor rhythm in WT animals (**Figure 4C1**) as well as the L2 burst amplitudes in SOD1 mice (**Figure 4C3**) computed in the presence of 50 μm DA were significantly enhanced when DA concentration was raised to 500 μm. In contrast, the other locomotor parameters tended to increase in the presence of 500 μm DA but were not significantly different from those computed in the presence of 50 μm DA (**Figure 4C**). These results indicate that DA neuromodulatory processes are similar in WT and SOD1 spinal motor networks. These results are in agreement with the HPLC analysis of spinal DA content that failed to reveal differences between WT and SOD1 mice at this developmental stage.

## DISCUSSION

### FICTIVE LOCOMOTION GENERATION IN SOD1 SPINAL NETWORKS

Our data show that in contrast to what has been previously described in the SOD1^G85R^ mouse line (Amendola et al., 2004), coordinated fictive locomotion could be efficiently triggered by the bath-application of NMA plus 5-HT in the SOD1^G93A^ mouse spinal cord. Several mutant SOD1 mouse models that serve as invaluable tools to understand the pathophysiology of ALS have been developed. To date, 12 lines of transgenic mouse expressing different human mutated SOD1 proteins are available. Transgenic SOD1^G93A^ mice are principally used in ALS research, followed by SOD1^G37R^, SOD1^G85R^, and SOD1^G86R^ mice. All of these models exhibit slightly different time courses of the disease and associated neurodegenerative processes depending on the SOD1 mutation site, related enzymatic activity, transgene copy number and genetic background (Jonsson et al., 2006; Turner and Talbot, 2008; Van Den Bosch, 2011). SOD1^G93A^ proteins, for example, are about 10 times more active than the native SOD1 proteins while in contrast, SOD1^G85R^ mutant proteins are almost inactive. Striking discrepancies between SOD1 models have been previously reported concerning motoneuron excitability (Meehan et al., 2010; Delestrée et al., 2014). Pambo-Pambo et al. (2009) have also shown that motoneurons are more immature in the *SOD1^G93A^* low expressor line (*SOD1^G93A^* ^low^) compared to *SOD1^G85R^.* Indeed, in newborn mice, SOD1^G93A^ ^low^ motoneurons have a more depolarized resting membrane potential and appear to be more excitable than SOD1^G85R^ and WT motoneurons. The difference between SOD1^G93A^ and SOD1^G85R^ mouse lines in NMA/5-HT’s effectiveness in triggering fictive locomotion further emphasizes the heterogeneity of SOD1 mouse mutants. This observed heterogeneity certainly parallels the complex etiology of ALS and stresses the importance of the complementary use of the different SOD1 mouse models to explore the different aspects of this motor disease.

In the present study, the NMA-5-HT-evoked rhythm was neither qualitatively nor quantitatively different between SOD1 and WT animals. These results suggest that whereas different groups have described early developmental alterations in motoneuron functioning, the locomotor outputs recorded from motoneuron axons in SOD1 mice are similar to the WT locomotor outputs. In the SOD1^G93A^ model, motoneurons at birth have been shown to be more immature, more excitable than WT motoneurons and to present a different dendritic branching pattern (Kuo, 2003; Pieri et al., 2003; Kuo et al., 2005; Pambo-Pambo et al., 2009). The question then arises as to whether these changes have a real impact on motoneuron output or whether the whole spinal network in charge of locomotion generation operates in such a way that these alterations are compensated. For example, it is well known that after a lesion, the spinal locomotor networks undergo a restructuring and can, after training, adapt their functioning and produce almost the same locomotor pattern that existed before the lesion (Barbeau and Rossignol, 1987; Rossignol et al., 2008). Such compensatory mechanisms may occur in newborn SOD1 spinal networks to ensure normal locomotor network function. A limitation of the present study is that locomotor activity was assessed using extracellular recordings. Further research is needed to further decipher the impact, at the cellular level, of the previously reported impairments in motoneuron excitability and morphology on the motor circuits.

### SPINAL MONOAMINERIGIC NEUROMODULATION

Our HPLC data of either whole or ventral half spinal samples show that the monoaminergic rates rose between birth and the second postnatal week in the lumbar enlargement. This result is in agreement with developmental studies that have reported a progressive rostrocaudal gradient of the monaminergic innervation associated with an increase in axonal density (Commissiong, 1983; Rajaofetra et al., 1989, 1992; Giménez y Ribotta et al., 1998; Pappas et al., 2008; Pearlstein, 2013). Interestingly, we report an increased content of DA in the whole lumbar spinal cord of P10 SOD1 mice compared to WT animals. As this discrepancy was not observed in ventral spinal cord samples, our results provide insights into changes in the DA contents in the dorsal part of the SOD1 lumbar cord. This area is densely innervated and controlled by dopaminergic pathways (for review see Millan, 2002). Significant damages in the sensory system have been described in the presymptomatic stages in SOD1 models (Guo et al., 2009; Filali et al., 2011). Altered sensorimotor development characterized by a delay in maturation processes has also been reported in newborn SOD1 mice (Amendola et al., 2004). The increased DA content in the SOD1 dorsal spinal cord found in this study may contribute to these early alterations. It has been shown that both a reduction or an amplification of the monaminergic spinal content leads to a delay in spinal circuit maturation (Nakajima et al., 1998; Cazalets et al., 2000; Vinay et al., 2002), suggesting that a precise intraspinal level of these compounds is required for normal spinal cord network development. In the present study, we observed that regardless of the mouse genotype, the three monoamines tested potentiate locomotor activity by boosting the amplitude of the ventral root bursts and increasing the locomotor period. While DA contents were increased in the P10 SOD1 lumbar spinal cords, we failed to report any modification in DA neuromodulation in SOD1 mice. This discrepancy could be explained by the fact that extracellular recordings were performed on spinal cord preparations from P1-P3 mice, ages where DA rates are similar between SOD1 and WT mice. The sustainable locomotor activities necessary for neuromodulatory studies are difficult to achieve in *in vitro* preparations from P10 mice (but see (Jiang et al., 1999). To assess the impact of the DA content increase on both basal membrane properties and DA sensitivity of the P10 lumbar motoneurons, patch-clamp recordings in spinal cord slices will be needed to investigate the effects of DA antagonists and agonists on these neurons. Dose-response curves will also have to be performed to assess and compare DA sensitivity in WT and SOD1 motoneurons.

It is generally acknowledged that changes in the period and/or phase relationships of the locomotor rhythm reflects effects on the locomotor CPG while modifications in the burst amplitudes are associated with changes in the motoneuron or last order interneuron excitability (Miles and Sillar, 2011). In SOD1 mice, the period and phase relationships of the rhythm expressed in the presence of the three monoamines tested were similar to those found in WT animals. The modulation of burst amplitude was also comparable between SOD1 and WT mice in the presence of 5-HT and DA. In contrast, the amplitude values of the bursts recorded in the presence of NA were more amplified in SOD1 mice compared to WT littermates. These results suggest that the monoaminergic neuromodulation of the locomotor CPG is preserved and normal in newborn SOD1^G93A^ mice but that SOD1 motoneurons exhibit an increased sensitivity to NA compared to WT motoneurons. As the spinal cord size is not different between SOD1 and WT mice (personal unpublished observation), this effect could not be explained by differences in NA penetration into and the final concentrations attained within the spinal tissue (see for example Brumley et al., 2007). Lumbar motoneurons express the α_1_, α_2a_, and β_1_ receptors at birth (Rekling et al., 2000; Tartas et al., 2010). We have previously shown in newborn rats that the activation of these receptors increased the lumbar motoneuron excitability partly via the inhibition of the inwardly rectifying K^+^ current, K_IR_, a key determinant of neuronal excitability (Tartas et al., 2010). In addition, NA, through the activation of presynaptic α1 and β receptors, enhanced the synaptic transmission originating from the upper lumbar segments in motoneurons (Tartas et al., 2010). Possible alterations in the expression of Kir channels and/or noradrenergic receptors could sustain part of the increased sensitivity to NA in SOD1 spinal cord by modifying both the intrinsic membrane properties of motoneurons and the excitatory synaptic inputs they receive. Up-regulation of NA receptor number, for example, could lead to NA supersensitivity in SOD1 motoneurons. This kind of phenomenon has been described for DA receptors in diverse pathological conditions (see for examples: Briand et al., 2008; Seeman, 2013).

Due to their lack of Ca^2+^ buffering proteins, motoneurons are more prone to excitotoxicity than other neurons. An excess of excitatory inputs has been hypothesized to play a major role in the neuronal degeneration observed in ALS (Turner et al., 2013). The NA hypersensitivity we reported in the present study could trigger aberrant depolarizations and subsequent Ca^2+^ entries in the lumbar motoneurons leading to progressive damage to the intracellular machinery. It is thus of interest to further decipher the cellular basis of the NA neuromodulation in newborn SOD1 motoneurons and to investigate the NA antagonist effects on the SOD1 mouse life span.

## Conflict of Interest Statement

The authors declare that the research was conducted in the absence of any commercial or financial relationships that could be construed as a potential conflict of interest.
